# The role of auditory and cognitive factors in understanding speech in noise by normal-hearing older listeners

**DOI:** 10.3389/fnagi.2014.00307

**Published:** 2014-11-12

**Authors:** Tim Schoof, Stuart Rosen

**Affiliations:** Speech, Hearing and Phonetic Sciences, University College LondonLondon, UK

**Keywords:** aging, speech perception, auditory processing, cognition, noise

## Abstract

Normal-hearing older adults often experience increased difficulties understanding speech in noise. In addition, they benefit less from amplitude fluctuations in the masker. These difficulties may be attributed to an age-related auditory temporal processing deficit. However, a decline in cognitive processing likely also plays an important role. This study examined the relative contribution of declines in both auditory and cognitive processing to the speech in noise performance in older adults. Participants included older (60–72 years) and younger (19–29 years) adults with normal hearing. Speech reception thresholds (SRTs) were measured for sentences in steady-state speech-shaped noise (SS), 10-Hz sinusoidally amplitude-modulated speech-shaped noise (AM), and two-talker babble. In addition, auditory temporal processing abilities were assessed by measuring thresholds for gap, amplitude-modulation, and frequency-modulation detection. Measures of processing speed, attention, working memory, Text Reception Threshold (a visual analog of the SRT), and reading ability were also obtained. Of primary interest was the extent to which the various measures correlate with listeners' abilities to perceive speech in noise. SRTs were significantly worse for older adults in the presence of two-talker babble but not SS and AM noise. In addition, older adults showed some cognitive processing declines (working memory and processing speed) although no declines in auditory temporal processing. However, working memory and processing speed did not correlate significantly with SRTs in babble. Despite declines in cognitive processing, normal-hearing older adults do not necessarily have problems understanding speech in noise as SRTs in SS and AM noise did not differ significantly between the two groups. Moreover, while older adults had higher SRTs in two-talker babble, this could not be explained by age-related cognitive declines in working memory or processing speed.

## 1. Introduction

Older adults often experience increased difficulties understanding speech in noisy environments (CHABA, 1988), even in the absence of hearing impairment (Dubno et al., 2002; Helfer and Freyman, 2008). One type of masker that seems particularly detrimental to older adults is competing speech (Tun and Wingfield, 1999; Helfer and Freyman, 2008; Rajan and Cainer, 2008). It has similarly been suggested that normal-hearing older adults benefit less from fluctuations in the masker compared to young adults (Takahashi and Bacon, 1992; Stuart and Phillips, 1996; Peters et al., 1998; Dubno et al., 2002, 2003; Gifford et al., 2007; Grose et al., 2009). It remains unclear, however, what is specific to aging, independent of hearing loss as defined in terms of the audiogram, that explains these difficulties in the perception of speech in noise.

One possible explanation is that speech in noise difficulties in part arise from an age-related auditory temporal processing deficit (e.g., Frisina and Frisina, 1997; Pichora-Fuller and Souza, 2003; Pichora-Fuller et al., 2007). A useful way to think about auditory temporal processing is in terms of the decomposition of sound in the time domain into a slowly varying envelope (ENV) superimposed on a more rapidly varying temporal fine structure (TFS) (Moore, 2008). Aging has in fact been associated with declines in both ENV and TFS processing. Age-related declines in ENV processing become apparent, for instance, in terms of increased amplitude-modulation (Purcell et al., 2004; He et al., 2008) and gap detection thresholds (Snell, 1997; Schneider and Hamstra, 1999). Similarly, support for an age-related decline in TFS processing comes from a variety of psychophysical measures, such as frequency modulation (FM) detection (He et al., 2007), pitch discrimination using harmonic and inharmonic complex sounds (Vongpaisal and Pichora-Fuller, 2007; Füllgrabe, 2013), and the detection of inter-aural phase or time differences (Pichora-Fuller and Schneider, 1992; Grose and Mamo, 2010). These temporal processing deficits, and ultimately the increased difficulties understanding speech in noise, may be the result of disrupted neural sound encoding that are manifest even in the absence of any elevation in audiometric thresholds (Pichora-Fuller et al., 2007; Anderson et al., 2012; Sergeyenko et al., 2013).

While it is reasonable to assume that envelope processing is particularly important for the perception of speech in fluctuating maskers, age-related declines in temporal envelope processing may not necessarily be the cause of the decreased fluctuating masker benefit (FMB) in older adults. For one, FMB may be reduced in older adults at relatively slow modulation rates (e.g., 10 Hz; Dubno et al., 2002, 2003; Gifford et al., 2007), while age-related declines in ENV processing only become apparent at higher modulation rates (above about 200 Hz, e.g., Purcell et al., 2004; Grose et al., 2009). Instead, older adults might simply be less able to make use of the information in the dips of the fluctuating masker (Grose et al., 2009).

A perhaps more compelling theory is that an age-related decline in TFS processing partly explains difficulties understanding speech in noise. It has been argued that while ENV information may be sufficient for the perception of speech in quiet (Shannon et al., 1995), TFS may be required to successfully understand speech in the presence of interfering sound sources (Lorenzi et al., 2006; Moore, 2008, 2012). Although it has previously been suggested that TFS may be important to benefit from amplitude dips in fluctuating maskers (Schooneveldt and Moore, 1987; Lorenzi et al., 2006; Moore, 2008), the role of TFS may not necessarily be in detecting glimpses, but it may instead allow efficient auditory scene analysis and/or spatial release from masking (Bernstein and Brungart, 2011; Moore, 2012). In other words, age-related declines in TFS processing could be equally important in accounting for difficulties in steady-state noises as well as those that fluctuate. Furthermore, age-related declines in TFS processing may particularly impact speech perception in the presence of competing talkers as TFS provides pitch cues for sound source segregation (e.g., Bregman, 1990; Darwin and Carlyon, 1995).

An alternative explanation is that speech in noise difficulties in part arise from age-related declines in cognitive processing. Aging is associated with declines in several cognitive abilities that are thought to be important for the perception of speech in noise, such as working memory, attention, and processing speed (Craik and Byrd, 1982; Cohen, 1987; Kausler, 1994; Salthouse, 1996). However, older adults may in fact require more cognitive resources, putting higher demands on top-down processing to interpret the speech signal in the presence of background noise. Such demands may increase further when the input signal is further degraded as a result of auditory temporal processing declines (Rönnberg et al., 2010).

Working memory capacity, which refers to the ability to simultaneously store and process task-relevant information (Daneman and Carpenter, 1980; Baddeley, 1986), is perhaps most important for the perception of speech in noise (Rönnberg, 2003; Akeroyd, 2008). In a review of 20 studies looking at the role of cognition in speech perception in noise, Akeroyd (2008) found that working memory capacity, especially as assessed by the reading span test (Daneman and Carpenter, 1980; Rönnberg et al., 1989), was most predictive of speech perception in noise. Given that working memory capacity decreases with age (e.g., Craik and Jennings, 1992; Van der Linden et al., 1994), it is not unreasonable to assume that a decline in working memory plays an important role in the difficulties older adults experience when understanding speech in noise (Pichora-Fuller et al., 1995).

Similarly, selective attention, the ability to focus on relevant information and ignore irrelevant information, is probably equally important for successful speech understanding, especially in the presence of competing talkers which requires the suppression of meaningful competing information. Older adults may be less successful, however, at ignoring competing talkers as a result of an age-related decline in executive function, and more specifically a decline in inhibitory control (Hasher and Zacks, 1988; Hasher et al., 1999).

Underlying these age-related changes in working memory and attention may be a decline in processing speed. Salthouse (1985, 1996) argued that age-related declines in cognitive function may be the result of “cognitive slowing.” A reduction in processing speed means that relevant operations cannot be executed successfully in the time available and that the amount of simultaneously available information required for higher level processing is reduced. An age-related decline in processing speed may thus in part explain the speech in noise difficulties (Schneider et al., 2010).

Another factor thought to be important for speech perception in noise is linguistic closure, a supra-modal linguistic capacity thought to reflect the ability to fill in missing information (c.f. Zekveld et al., 2007). Linguistic closure is often assessed using the Text Reception Threshold (TRT), a visual analog of the SRT task, in which participants read sentences masked by bars of varying widths. This task was developed, more generally, to assess the extent to which inter-individual differences in speech in noise performance can be attributed to non-auditory factors. It remains unclear, however, whether the ability to read masked text decreases with age (see Besser et al., 2013, for a review).

The aim of this study was to assess why older adults, even in the absence of hearing impairment, experience increased difficulties understanding speech in noise. This study is novel in two ways. Firstly, relatively strict criteria for normal hearing were used (thresholds <25 dB HL up to 6 kHz). Secondly, while the majority of studies examining the effects of aging on speech perception in noise have used simple target stimuli, such as syllables (e.g., Stuart and Phillips, 1996; Dubno et al., 2002) or simple sentences (e.g., Peters et al., 1998; Gifford et al., 2007), this study used more complex targets (IEEE sentences; Rothauser et al., 1969)

Speech perception was assessed in the presence of different types of background noise. First, to examine whether normal-hearing older adults indeed benefit less from amplitude fluctuations in the masker, speech reception thresholds (SRTs) were measured in steady-state and amplitude-modulated noise (c.f. Takahashi and Bacon, 1992; Stuart and Phillips, 1996; Peters et al., 1998; Dubno et al., 2002, 2003; Gifford et al., 2007; Grose et al., 2009). Second, SRTs were also measured in the presence of two-talker babble since competing speech is both ecologically valid and particularly detrimental for older adults (Tun and Wingfield, 1999; Helfer and Freyman, 2008; Rajan and Cainer, 2008). In addition, various measures of auditory temporal (ENV and TFS) and cognitive processing (working memory, attention, processing speed, linguistic closure, and reading skills) were assessed to examine the relative contribution of declines in both domains on speech perception difficulties.

Individual differences in cognitive processing appear to be the most important factor explaining aided speech understanding in noise, after accounting for differences in audiometric thresholds, for hearing impaired older adults (see reviews by Humes et al., 2007; Akeroyd, 2008; Houtgast and Festen, 2008; Humes and Dubno, 2010). Therefore, age-related cognitive declines may also be expected to be the primary contributor to increased difficulties in speech perception in noise for normal-hearing older adults.

## 2. Materials and methods

### 2.1. Participants

**Figure 1:**
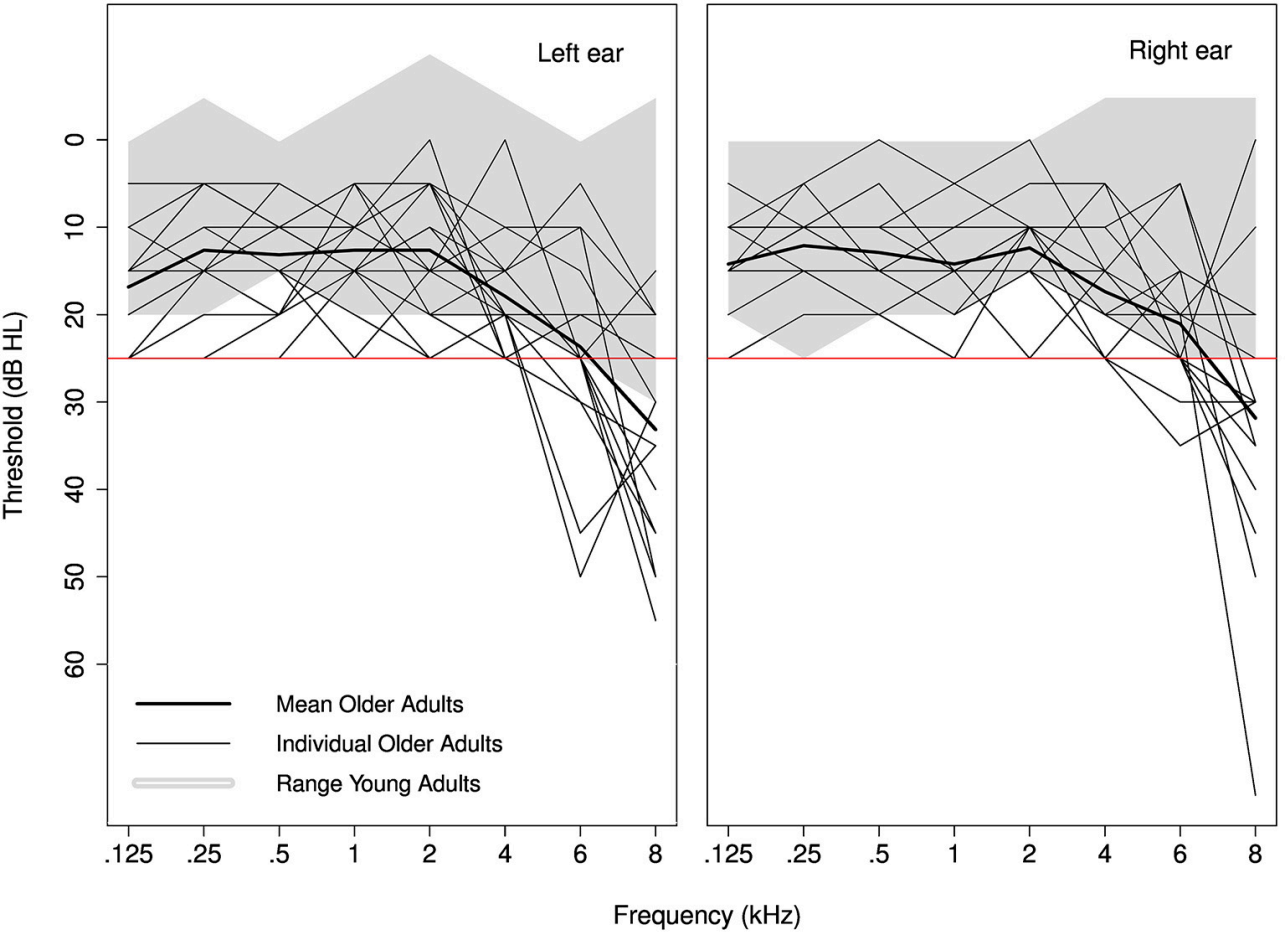
**Individual audiograms for older adults are plotted for the left and right ear separately**. The shaded area represents audiometric thresholds of the younger adults. The red line indicates the inclusion criterion of 25 dB HL.

Nineteen young (19–29 years old, mean 23.7 years, *SD* 2.9 years, 10 males) and 19 older (60–72 years old, mean 64.1 years, *SD* 3.3 years, 3 males) monolingual native English speakers participated in this study. All participants had near-normal hearing defined as (air-conducted) pure-tone thresholds of 25 dB HL or better at octave frequencies from 0.25 to 4 kHz in both ears and at 6 kHz in at least one ear (Figure 1). In addition, all participants over the age of 65 had normal cognitive function [scores ≥17 MMSE telephone version (Roccaforte et al., 1992)] and normal or corrected-to-normal vision. None of the participants reported a history of language or neurological disorders. All participants signed a consent form approved by UCL Research Ethics Committee and were paid for their participation.

### 2.2. Speech perception in noise

Speech reception thresholds (SRT) were measured for sentences in different types of background noise. The target stimuli were pre-recorded IEEE sentences (Rothauser et al., 1969) produced by a male talker with a standard Southern British accent. Each sentence contained five keywords. The sentences were presented in steady-state speech-shaped noise (SS), speech-shaped noise sinusoidally amplitude modulated at 10 Hz (AM) with a modulation depth of 100%, and two-talker babble [see Rosen et al. (2013), for a description of the speech-shaped noise and two-talker babble]. The masker always started 600 ms prior to stimulus onset and was gated on and off across 100 ms.

To rule out possible contributions of differences in audiometric thresholds above 6 kHz, the stimuli were low-pass filtered at 6 kHz using a 4th order Butterworth filter. In addition, for six older participants with thresholds >25 dB HL at 6 kHz in one ear the stimuli were spectrally shaped using the National Acoustics Laboratories-Revised (NAL-R) linear prescriptive formula based on their individual thresholds (Byrne and Dillon, 1986).

The participants were seated in a soundproof booth and listened to the stimuli over Sennheiser HD 25 headphones. They were asked to repeat verbatim what they heard. The experimenter scored responses using a graphical user interface (GUI) which showed the five key words. The scoring screen was not visible to the participants and no feedback was provided.

The SNR was varied adaptively following the procedure described by Plomp and Mimpen (1979). The first sentence was presented at an SNR of −10 dB. Until at least 3 out of 5 key words were correctly repeated, the SNR was increased by 6 dB on the next presentation. The initial sentence was repeated until at least 3 out of 5 keywords were repeated correctly or the SNR reached 30 dB. For each subsequent sentence the SNR increased by 2 dB when 0–2 key words were correctly repeated or decreased by the same amount for 3–5 correct repetitions. The number of trials was fixed at twenty, tracking 50% correct.

SRTs for each condition were measured twice. A measurement was repeated, with a different set of sentences, when fewer than 3 reversals were obtained or when the standard deviation across the final reversals exceeded 4 dB. Thresholds for each run were computed by taking the mean SNR (dB) across the reversals at the final step size of 2 dB.

Participants were given brief training on the different conditions to familiarize them with the different types of background noise. Practice consisted of 5 trials and started at 0 dB SNR. The order of conditions in the experiment proper was counterbalanced across participants following a Latin square design. Stimuli were presented binaurally at 70 dB SPL.

### 2.3. Subjective measure of speech perception in noise

Participants were asked to complete section one of the Speech, Spatial, and Qualities of Hearing Scale (SSQ; Gatehouse and Noble, 2004), which addresses listeners' abilities to understand speech in quiet as well as in the presence of different types of noise. Composite scores were calculated for each participant by averaging across all questions.

### 2.4. Temporal processing

Participants completed three tasks that assess temporal processing; gap detection, amplitude modulation (AM) detection and frequency modulation (FM) detection. While the gap and AM detection tasks are concerned with temporal resolution in the envelope domain, the FM detection task assesses processing of TFS. The general procedure was similar for all three tasks. More details on the different tasks are provided below.

In all three tasks, a 3AFC paradigm was used and participants were asked to identify the stimulus that either contained a gap, or was modulated in amplitude or frequency. The duration of the gap or the depth of modulation was varied adaptively following the adaptive three-down, one-up procedure thus tracking 79% (Levitt, 1971).

Thresholds were obtained across two runs. A run was terminated after six reversals or after a maximum of 50 trials. Thresholds were computed by taking the mean gap duration or modulation depth across the last four reversals of each run. Thresholds reported here are the mean across the two runs.

Participants received training on five trials to familiarize themselves with the task. During this brief training they received visual feedback. During the experiment proper no feedback was provided.

Stimuli were presented binaurally over Sennheiser HD 25 headphones at 70 dB SPL. The order of the three tasks was counterbalanced across participants following a Latin square design.

#### 2.4.1. Gap detection

Gap detection thresholds were measured using three 3-kHz-wide noises bandpass filtered between 1 and 4 kHz. A relatively wide band of noise was used as this limits the confounding effect of inherent fluctuations of the noise source on gap detection thresholds. The stimuli had a duration of 400 ms with a 10 ms rise-fall time and an inter-stimulus interval of 500 ms. The bands of noise were generated online at the start of each trial. All three noise bursts were thus based on the same underlying 400 ms section of noise. When a temporal gap was present in the stimulus, it was centered 300 ms after stimulus onset. Gap durations were varied from 0.5 to 7 ms in 20 logarithmic steps. Gaps were created by zeroing the waveform. Since this results in spectral cues that could aid the listener in identifying the presence of a gap, the stimuli were filtered to the required bandwidth after the insertion of the gap using a 4th order Butterworth filter. It should be noted that this procedure causes some temporal smearing of the gap. However, for relatively shallow filters this should not affect gap detection thresholds too much (c.f. Eddins et al., 1992).

The initial gap duration was 7 ms and was decreased after each trial until an error was made. Subsequently, three consecutive correct responses were required to decrease the gap duration, while one incorrect response increased the gap duration. The initial step size was 3 logarithmic steps and was decreased to 2 and finally 1 logarithmic step after each reversal. To prevent the gap duration from decreasing too far below the participant's threshold during the first few runs, the step size was automatically set to 1 logarithmic step once the gap duration was ≤1 ms. A run was repeated when fewer than 3 reversals were obtained or when the standard deviation across the final reversals exceeded 2 ms.

#### 2.4.2. AM detection

As in the gap detection task, AM detection thresholds were measured using three 3-kHz-wide noises bandpass filtered between 1 and 4 kHz. The temporal-modulation transfer function was determined on the basis of AM detection thresholds for five (sinusoidal) AM rates: 10, 20, 40, 80, and 160 Hz. These modulation rates are all multiples of 10 Hz, which is the modulation rate of the masker used in the speech perception in noise task. The duration of the stimuli was 500 ms, which resulted in a whole number of AM cycles in all four conditions. The stimuli had a 10 ms rise-fall time and a 500 ms inter-stimulus interval. As in the gap detection task, the bands of noise were generated online at the start of each trial, which meant that the three stimuli in each trial were composed of the same noise sample. Amplitude modulation depths varied in 25 steps of 1 dB from −8 to −32 dB for rates up to 80 Hz and from −5 to −29 dB for the 160 Hz modulation rate. Since AM of bandpassed noise produces spectral side bands, the stimuli were filtered using a 4th order Butterworth filter after modulation. It should be noted that this may have reduced the effective modulation depth, especially for higher AM rates, although the filtering used should not have much of an effect (c.f. Eddins, 1993, 1999).

On the initial trial, the modulation depth was set to −8 dB, or −5 dB for the 160 Hz modulation rate, and was decreased after each trial until the participant gave an incorrect response. Subsequently, three consecutive correct responses were required to decrease the AM depth, while one incorrect response increased the AM depth. The initial step size was 6 dB, and was decreased in four steps after each reversal to the final step size of 1 dB. To prevent the AM depth from overshooting the participant's threshold during the initial runs, the step size was automatically set to 1 dB once the AM depth reached ≤−25 dB for modulation rates of 10 and 160 Hz, and ≤−20 dB for modulation rates of 20, 40, and 80 Hz. A run was repeated when fewer than 3 reversals were obtained or when the standard deviation across the final reversals exceeded 3 dB. The order of conditions was counterbalanced across participants following a Latin square design.

Since the temporal modulation transfer function (TMTF) resembles the form of a low-pass filter (Viemeister, 1979), the AM detection thresholds were fitted with an equation describing the frequency response of a low-pass Butterworth filter using a non-linear least-squares regression (Eddins, 1993):

(1) $$y=10log_{10}\left(\frac{1}{1+{\left(\alpha f\right)}^{2}}\right)+c$$

where y is the gain of the imputed filter (in dB) and f is the modulation rate in Hz. The inverse of α gives the −3 dB cutoff frequency (TMTF cutoff frequency) and c (the y-intercept) provides a measure of efficiency (AM efficiency). Note that a higher α (i.e., a higher cutoff frequency) and a lower c (i.e., better efficiency) indicate better performance.

#### 2.4.3. FM detection

FM detection thresholds were determined using a 1 kHz sinusoidal carrier modulated at 2 Hz. A relatively low carrier frequency and modulation rate were used to ensure participants could only detect FM based on temporal cues (Moore and Sek, 1995, 1996). Frequency modulation depths varied logarithmically between 0.02 and 4.5 dB in 30 steps. The stimuli had a duration of 1 s, which is equal to 2 FM cycles. The interstimulus interval was set to 500 ms.

On the initial trial the modulation depth was set to 4.5 dB and was decreased after each trial until the listener made an error. Subsequently, three consecutive correct responses were required to decrease the FM depth, while one incorrect response increased the FM depth. The initial step size was three logarithmic steps, and was decreased in three steps after each reversal to the final step size of one logarithmic step. In addition, the step size was automatically set to one logarithmic step once the FM depth reached ≤0.57 dB to prevent the FM depth from overshooting the participant's threshold during the initial runs. A run was repeated when fewer than 3 reversals were obtained or when the standard deviation across the final reversals exceeded 2 dB.

FM detection thresholds are reported as modulation indices, which is the modulation depth divided by the modulation rate (2 Hz).

### 2.5. Cognitive skills

Cognitive skills were assessed in the visual domain to ensure that auditory factors did not influence these measures.

#### 2.5.1. Working memory

A reading span task was used to examine participants working memory capacity (Rönnberg et al., 1989). This task was designed to tax not only information storage and rehearsal (as do, for example, digit span and word span tasks) but also information processing. The reading span task developed by Rönnberg and colleagues is an extension of the task developed by Daneman and Carpenter (1980). Here, participants were asked to read sequences of 3–6 three-word sentences and judge whether the sentence was semantically sensible or not (e.g., “The train sang a song,” or “The girl brushed her teeth”). At the end of each sequence of sentences, participants were asked to recall either the first or last word of each sentence in the correct order. The typeface of the text was Helvetica with font size 40. Words were presented in black on a gray background at 0.8 s/word. The inter-sentence interval, during which participants are required to make a semantic judgment, was 1.75 s. Participants were given one sequence of three sentences as a practice trial. During the testing phase, participants were presented with three runs of each sequence length (i.e., 3–6 sentences). The number of correctly remembered words was recorded.

#### 2.5.2. Attention

Participants were assessed on the Visual Elevator task, a subtask of the Test of Everyday Attention (TEA; Robertson et al., 1996). It is thought to reflect an ability to switch attention, which is important for understanding speech in noise, especially in the presence of competing talkers. In essence, the participants' task was to count in a certain direction and at a given cue start counting in the opposite direction. The task consists of 10 trials. Participants were asked to determine the floor number for each item and complete the task as fast as they could. The responses for each trial and the total time required to complete all 10 trials were recorded. The total number of reversals for all correct responses were subsequently recorded. The final score was calculated by dividing the total duration required to complete the task (in seconds) by the total number of reversals for the correct responses.

#### 2.5.3. Processing speed

To assess processing speed, participants were asked to complete the Letter Digit Substitution Test (LDST; Van der Elst et al., 2006). Participants were asked to complete the written version of the LDST. They were provided with a key in which the numbers 1–9 are each paired with a different letter. The test items, consisting of eight rows of 15 randomized letters, were printed below the key. The letters and digits were printed in font size 14. None of the participants had difficulties reading the items. The participants were asked to replace the letters by the corresponding digits as quickly as possible in sequential order. The first 10 items were practice items. After completion of the test items they were given 60 s to substitute as many items as possible. The score is the number of correctly substituted items. Note that potential age-related declines in motor performance were not controlled for.

#### 2.5.4. Text reception threshold

The text reception threshold (TRT) is a visual analog of the speech reception threshold (SRT), especially in fluctuating noise (Zekveld et al., 2007; Besser et al., 2012). This task was developed to measure the variance in speech perception in noise abilities that are associated with supra-modal cognitive and linguistic skills. In this task sentences that are partly masked by a vertical bar pattern are presented on a computer screen.

As in the speech perception in noise task (measuring SRTs), the target stimuli were IEEE sentences (Rothauser et al., 1969). While the target stimuli were taken from the same corpus, the specific sentences used in the two tasks were different. The participants were seated approximately 50 cm from the screen. The typeface used to present the sentences was Arial, with a font size of 28. The background color was white, the masked bar pattern was black, and the sentences were presented in red. The participants were asked to read the sentence out loud. The experimenter scored responses using a graphical user interface (GUI) which showed all the words in the sentence. The scoring screen was not visible to the participants and no feedback was provided.

The degree of masking was varied adaptively following the procedure described by Plomp and Mimpen (1979). The first sentence was presented with 16% unmasked text. Until the sentence was correctly repeated, the percentage of unmasked text was increased by 12% on the next presentation. Subsequent sentences were only presented once. When a sentence was correctly repeated, the degree of masking was increased by 6%. Conversely, the degree of masking was decreased by 6% when a sentence was not repeated correctly, thus tracking 50% correct.

TRTs were measured in response to two lists of twenty sentences each. Thresholds for each run were computed by taking the mean percentage unmasked text across sentences 5–20. The thresholds reported here are the mean across the two trials.

#### 2.5.5. Reading skills

Given that both the text reception threshold and the reading span tasks rely heavily on reading, participants were assessed on reading ability using the Test of Word Reading Efficiency (TOWRE, Torgesen et al., 1999). Participants were asked to read out a list of 104 English words as fast as they could. Subsequently, they were asked to do the same for a list of 84 non-words. The words were presented in Arial font size 20. While the first subtask assesses participants' sight reading skills, the second subtask addresses their phonemic decoding efficiency. The TOWRE is aimed at children and normally assesses the number of words that can be correctly identified within 45 s. However, to avoid any ceiling effects in adults, participants read out all the words on the list and reading ability was assessed in terms of the time it took them to read the whole list. The score for this task was calculated by dividing the total duration required to complete the task by the number of correctly read items.

## 3. Results

Data points that fell outside the mean ±3 *SD* were considered outliers and excluded from the analyses reported below. In total, ten data points were excluded [data points from the older group were excluded for AM detection threshold at 160 Hz (one), TMTF cut-off (one), AM efficiency (one), TEA (two), TRT (one); data points from the young group were excluded for AM detection threshold at 160 Hz (one), TEA (two), and non-words TOWRE (one)].

Descriptive statistics for all measures as well as confidence intervals for the group differences are summarized in Table 1.

**Table 1:** **Descriptive statistics**.

**Dependent variable**	**Mean (*SD*) young**	**Mean (*SD*) older**	**CI**
SRT SS	−3.7 (1.5)	−3.8 (1.2)	(−1.0, 0.8)
SRT AM	−6.7 (2.4)	−6.4 (1.2)	(−1.0, 1.5)
SRT babble	−1.6 (2.0)	−0.3 (0.8)	(0.4, 2.4)
SSQ	7.5 (1.0)	7.1 (1.2)	(−1.2, 0.3)
AM cutoff	110 (50)	111 (62)	(−36, 38)
AM efficiency	−19.4 (2.0)	−19.5 (1.0)	(−1.2, 0.9)
FM detection	1.0 (0.4)	1.1 (0.3)	(−0.2, 0.3)
Gap detection	4.3 (0.8)	4.6 (1.2)	(−0.4, 0.9)
Working memory	32 (5.6)	24 (5.0)	(−11.6, −4.6)
Attention	4.2 (1.9)	5.0 (1.7)	(−0.4, 2.0)
Processing speed	39 (6.7)	34 (6.6)	(−9.3, −0.3)
TRT	63 (4.3)	63 (3.5)	(−1.8, 3.2)
Reading words	0.49 (0.09)	0.48 (0.07)	(−0.06, 0.05)
Reading non-words	0.74 (0.14)	0.72 (0.15)	(−0.1, 0.08)

*Descriptive statistics (mean and SD) for the young and older adults separately as well as confidence intervals for the group differences are provided for all measures*.

### 3.1. Audiometric thresholds

While both groups had near-normal hearing, defined as pure-tone thresholds ≤25 dB HL up to 4 kHz in both ears and at 6 kHz in at least one ear, their thresholds were significantly different. Independent *t*-tests indicated that pure-tone averages (PTA) across 0.5–4 kHz (all ≤25 dB HL) were significantly higher (i.e., worse) by 7.1 dB for the older age group [*t*_(36)_ = −6.4, *p* < 0.001]. This could potentially contribute to any group differences that might exist for the auditory tasks (SRT, gap detection, AM detection, and FM detection; see Section 3.6).

Analyses were conducted on a PTA across 0.5–4 kHz since the auditory tasks in this study, with the exception of the SRT task, did not have energy above 4 kHz. While the materials in the SRT task did contain energy above 4 kHz, stimuli for six older adults were spectrally shaped using the NAL formula to account for audibility differences.

### 3.2. Speech perception in noise

**Figure 2:**
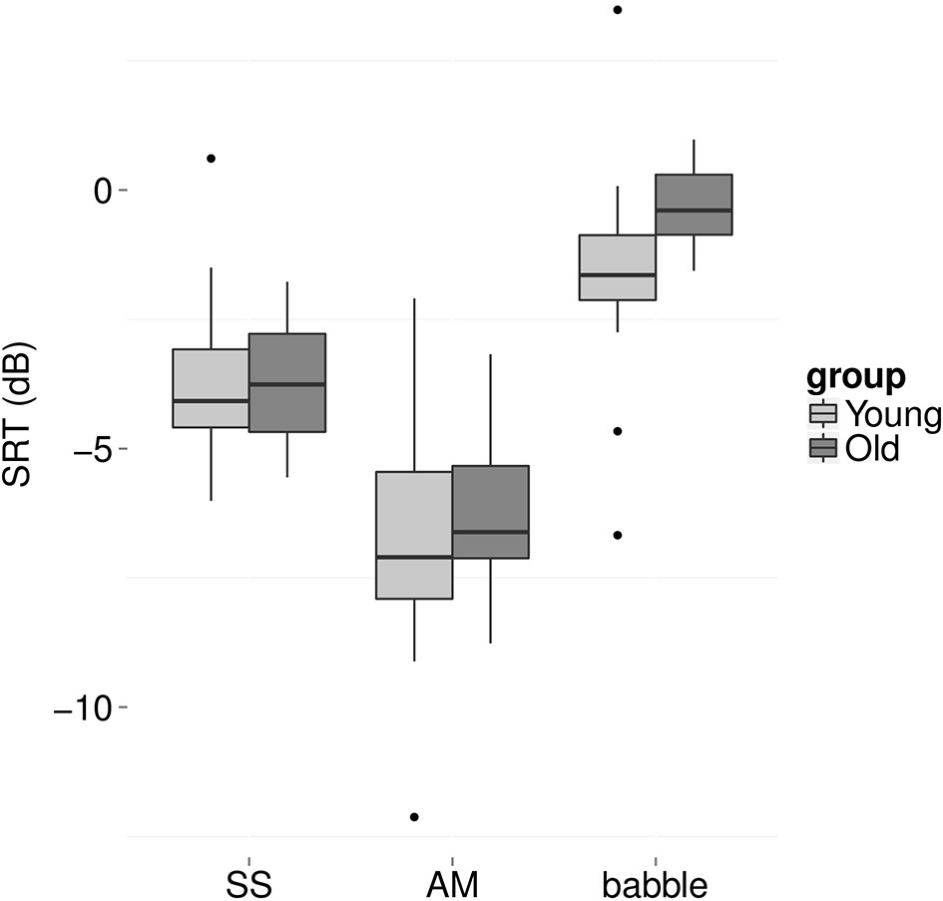
**Boxplots of speech reception thresholds (SRT, in dB) for young (light gray) and older (dark gray) listeners for SS noise (left), AM noise (middle), and two-talker babble (right)**.

Older adults were expected to perform more poorly (i.e., higher SRTs) in all three background noises. However, the older adults had higher SRTs only in the presence of two-talker babble (Figure 2). A mixed effects model with condition (AM, SS, babble) and group (young, old) as fixed factors and participant and sentence list as random factors showed a significant interaction between condition and group [*F*_(2, 186)_ = 5.6, *p* = 0.004]. *Post-hoc* independent *t*-tests revealed a significant difference between the two age groups for babble only, with young listeners performing better than older listeners by 1.4 dB [*t*_(36)_ = 2.8, *p* = 0.008, Cohen's *d* = 0.9; all other *p* > 0.6].

Overall, SRTs in AM noise were expected to be lower (i.e., better) compared to SRTs in SS noise, indicative of dip listening. Furthermore, SRTs in babble were expected to be higher (i.e., worse) compared to the two noise maskers (c.f. Rosen et al., 2013). *Post-hoc* independent *t*-tests indeed revealed a significant dip listening effect, with lower SRTs in AM compared to SS noise [*t*_(37)_ = 12.9, *p* < 0.001, Cohen's *d* = 1.4, mean difference = 2.7 dB]. In addition, SRTs in babble were significantly higher compared to the two noise maskers [SS: *t*_(37)_ = 8.5, *p* < 0.001, Cohen's *d* = 2.5, mean difference = 2.6 dB; AM: *t*_(37)_ = 16.3, *p* < 0.001, Cohen's *d* = 1.4, mean difference = 5.3 dB].

While there may be no group differences in SRTs in SS or AM noise and only a small difference in babble, it may be the case that particular older adults experience increased difficulties with one or more of the maskers. To explore these individual differences, we performed a deviance analysis (c.f. Ramus et al., 2003). The SRT scores were converted to z-scores and the deviance threshold was set to 1.65 *SD* above the mean SRT of the young group. Thus, participants were identified who performed more poorly than the poorest 5% of a young population.

**Figure 3:**
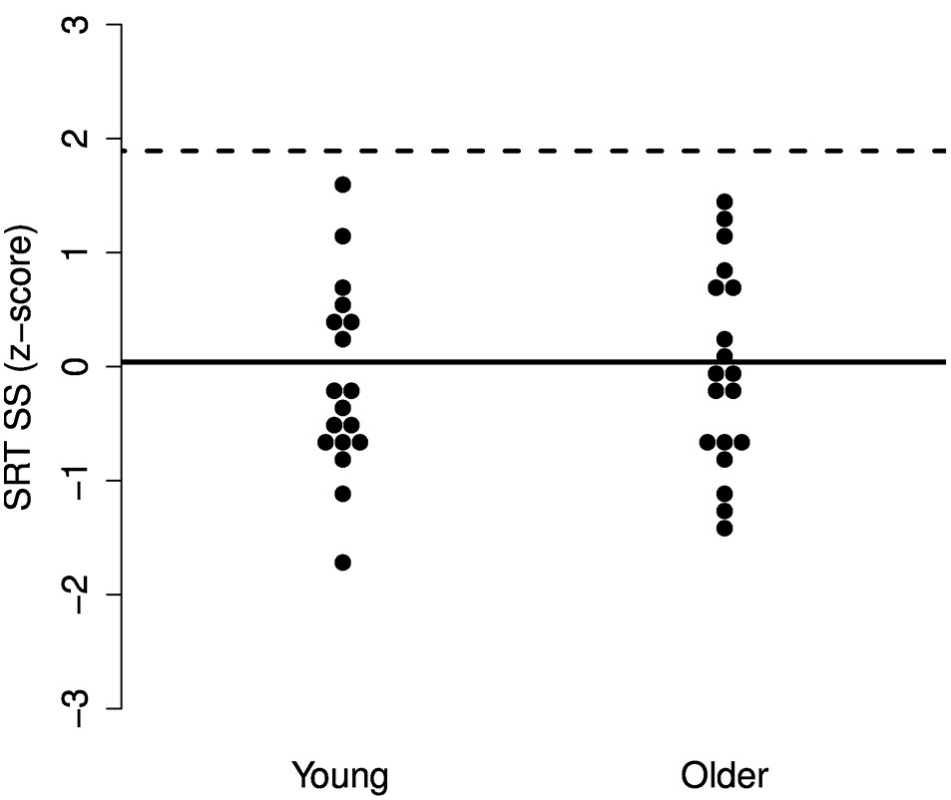
**Individual z-scores for the SRTs in SS noise**. The solid line indicates the mean for the young adults and the dotted line indicates the deviance threshold (1.65 *SD* above the mean for the young adults). No deviant older adults were identified.

**Figure 4:**
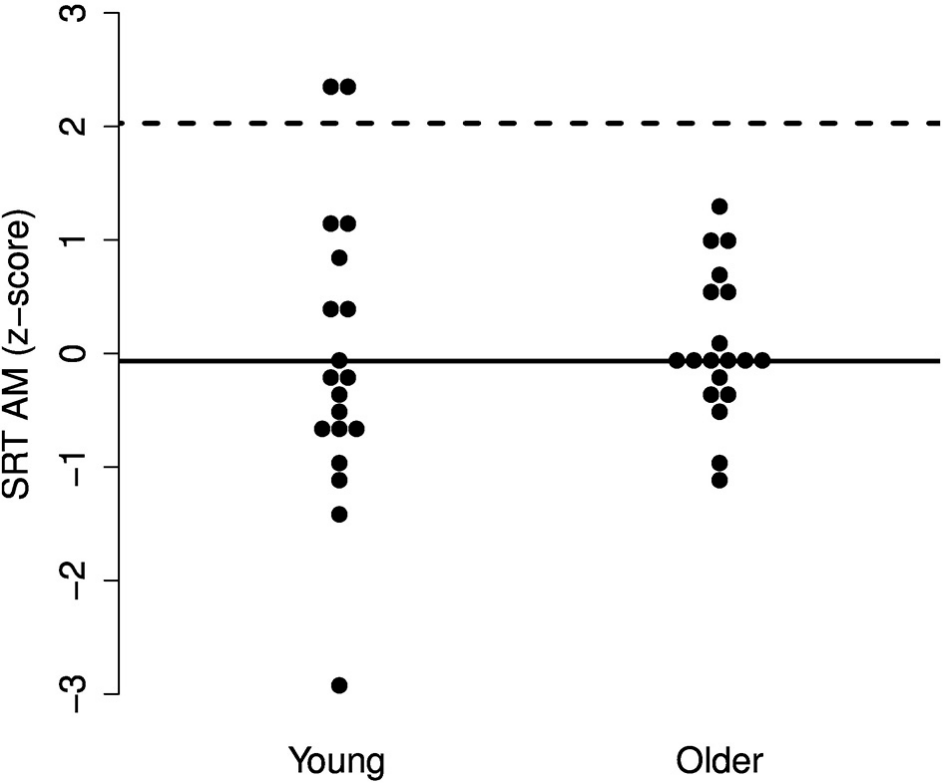
**Individual z-scores for the SRTs in AM noise**. The solid line indicates the mean for the young adults and the dotted line indicates the deviance threshold (1.65 *SD* above the mean for the young adults). No deviant older adults were identified.

**Figure 5:**
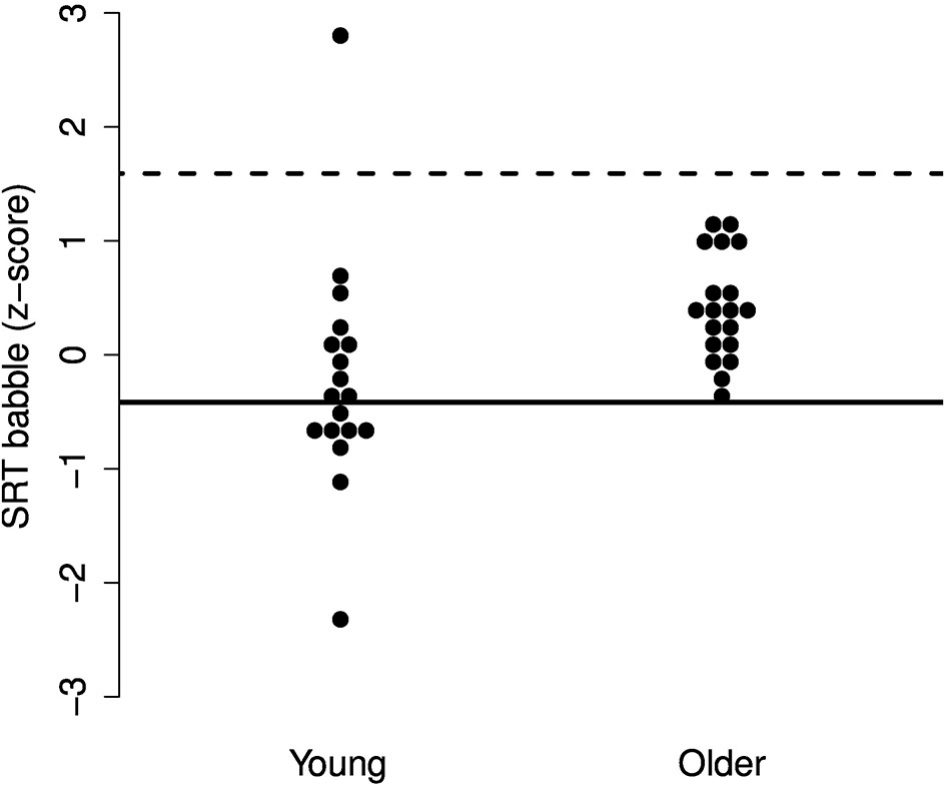
**Individual z-scores for the SRTs in two-talker babble**. The solid line indicates the mean for the young adults and the dotted line indicates the deviance threshold (1.65 *SD* above the mean for the young adults). No deviant older adults were identified.

The results, illustrated in Figures 3–5, indicate that none of the older adults performed particularly poorly in any of the maskers. This supports the idea that normal-hearing older adults do not necessarily experience increased difficulties understanding speech in noise.

### 3.3. Subjective measure of speech perception in noise

While the SRT data showed some group differences (in the presence of two-talker babble only), older adults did not report increased difficulties understanding speech in noise. An independent *t*-test on the subjective measure of speech perception in noise (SSQ questionnaire) did not reveal a significant difference between the two age groups [*t*_(36)_ = 1.3, *p* = 0.2]. It should be pointed out, however, that the difference in SRTs in two-talker babble was small (1.4 dB) and that older adults did not perform more poorly in AM and SS noise compared to the young adults.

### 3.4. Auditory temporal processing

While previous studies have reported age-related declines in auditory temporal processing (Pichora-Fuller and Schneider, 1992; Snell, 1997; Vongpaisal and Pichora-Fuller, 2007; He et al., 2008; Füllgrabe, 2013), no support for such a deficit was found in this study. AM, FM, and gap detection thresholds did not differ significantly between the young and older adults.

Independent *t*-tests on the two measures derived from the TMTF (AM efficiency and TMTF cut-off frequency) revealed no significant group differences [AM efficiency: *t*_(35)_ = −0.23, *p* = 0.8; TMTF cut-off: *t*_(35)_ = −0.07, *p* = 0.9].

These findings were supported by a mixed effects model on the AM detection thresholds with rate (10, 20, 40, 80, and 160) and group (young, old) as fixed factors and participant as a random factor. The analysis revealed a significant main effect of rate [*F*_(1, 148)_ = 220, *p* < 0.001], due to the fact that the shape of the TMTF resembles a low-pass filter. However, no group or interaction effects were found [group *F*_(1, 36)_ = 0.4, *p* = 0.5; interaction *F*_(1, 148)_ = 1.2, *p* = 0.27], which means that the AM detection thresholds at the five different rates did not differ between the young and older adults.

Similarly, independent *t*-tests did not reveal significant differences between the two age groups in terms of FM and gap detection thresholds [FM *t*_(36)_ = 0.6, *p* = 0.5; gap *t*_(36)_ = 0.7, *p* = 0.4].

### 3.5. Cognitive processing

**Figure 6:**
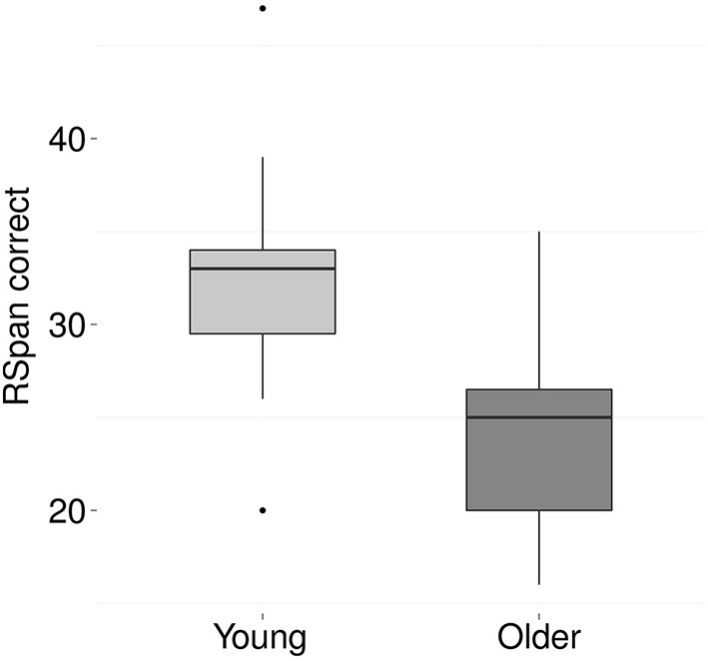
**Boxplots of the total number of correctly recalled words on the Reading Span test for young (light gray) and older (dark gray) participants**. On average, the young adults remembered 32 words (*SD* 5.5) and the older adults 23.9 words (*SD* 5).

**Figure 7:**
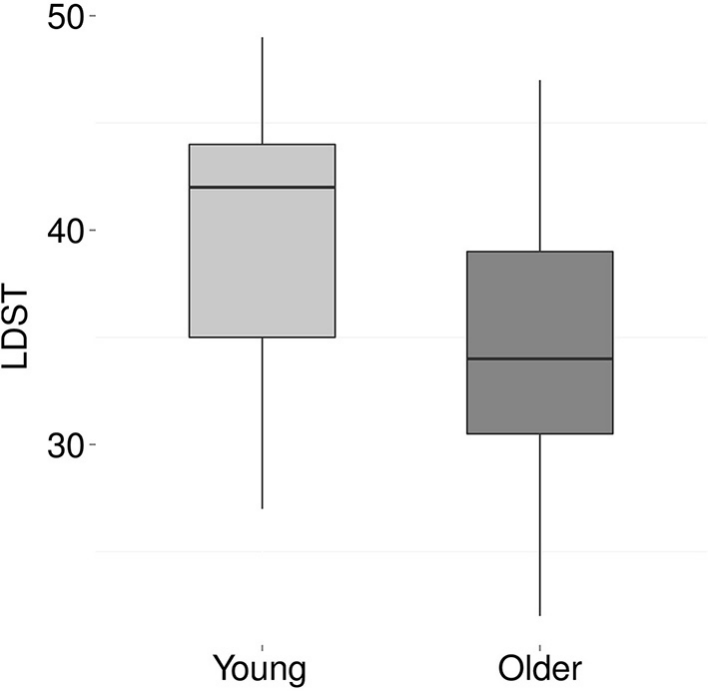
**Performance on the LDST task, reflecting processing speed, for young (light gray) and older (dark gray) participants**. Scores are the number of correctly substituted items in 60 s. The young adults substituted, on average, 39 items (*SD* 6.9) while the older adults only substituted 34 items (*SD* 6.6).

Figures 6, 7 show the results for the different cognitive processing tasks. Five independent *t*-tests were carried out to examine the effect of age on various cognitive skills. The analyses revealed an age-related decline in working memory, as indicated by fewer correctly remembered items on the Reading Span task [*t*_(36)_ = 4.7, *p* < 0.001, Cohen's *d* = 1.5]. In addition, a significant age-effect was found for processing speed, with older adults performing fewer substitutions on the letter-digit-substitution task [*t*_(36)_ = 2.2, *p* = 0.04, Cohen's *d* = 0.7]. No age-effects were found for attention [*t*_(32)_ = −1.3, *p* = 0.2], TRT [*t*_(35)_ = −0.6, *p* = 0.59], or reading skills [words *t*_(36)_ = 0.3, *p* = 0.8; non-words *t*_(35)_ = 0.4, *p* = 0.7].

### 3.6. Predicting speech perception in noise

Of primary interest was the extent to which the various auditory and cognitive measures could predict listeners abilities to perceive speech in the three noises. The results have so far indicated age-related declines in speech perception in babble (but not SS and AM noise), working memory, and processing speed. In addition, while both groups had near-normal hearing, thresholds for the older adults were significantly higher. These findings indicate that the normal-hearing older adults had no problems understanding speech in SS and AM noise, despite some age-related cognitive declines and slightly higher audiometric thresholds. One of the questions that remains, however, is whether these age-related declines can account for the group difference in SRTs in babble.

Furthermore, the fact that the older adults only experienced increased difficulties understanding speech in two-talker babble, but not in the two noise maskers (SS and AM noise), suggests that the relative contribution of the various auditory and cognitive processes involved in the perception of speech in noise differs depending on the masker type. A question to be answered, then, is which of the auditory and cognitive measures can account for the inter-individual differences in the perception of speech in the presence of babble and noise maskers.

**Figure 8:**
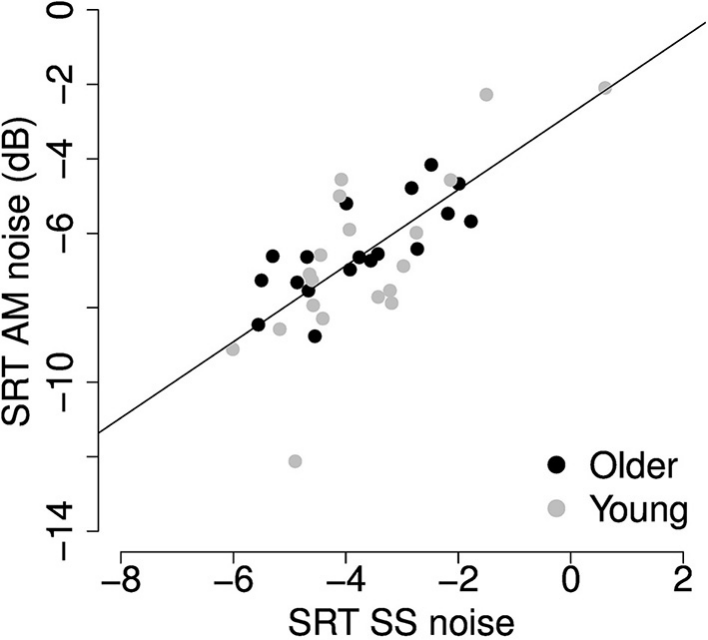
**Scatter plot of SRTs in SS and AM noise reveals a strong correlation (*r* = 0.736, *p* < 0.001) between performance in the two noise maskers**.

To determine which of the auditory or cognitive measures was predictive of speech understanding in babble and noise maskers, best subsets regression analyses were conducted (Hastie et al., 2009). Since the SRTs in AM and SS noise were highly correlated (*r* = 0.736, *p* < 0.001, *R*^2^ = 0.54, Figure 8), the regression was performed on the average of the two.

#### 3.6.1. Data reduction

Due to the relatively large number of possible predictors (twelve) given our sample size (38 participants), a principal components analysis (PCA) using varimax rotation with Kaiser normalization was performed on the cognitive and temporal processing tasks separately to reduce the number of predictors for the regression analysis. Missing data points (see Section 3) were replaced by the mean. The resulting principal components (PC) were saved as Anderson-Rubin scores to ensure uncorrelated PC scores.

PCA on the six cognitive measures (LDST, RSpan, TRT, TEA, and TOWRE words and non-words) resulted in the extraction of two components, following the Kaiser criterion (eigenvalues >1). Together they explained 63% of the variance in the data, with PC1 accounting for 34% and PC2 for 29% (see Table 2). The first PC was interpreted as an overall measure of linguistic closure (c.f. Zekveld et al., 2007) as it mainly reflected the TRT and the two measures of reading ability (TOWRE). The second PC primarily reflected processing speed (LDST) and working memory (RSpan). Note that the measure of attention (TEA) did not group clearly with either of the two components.

**Table 2:** **PCA Faor loadings: Cognitive processing**.

	**Linguistic closure**	**Memory and processing speed**
LDST	−0.187	**0.763**
Reading span	0.068	**0.857**
TEA	0.356	−0.518
TRT	**0.635**	−0.394
TOWRE words	**0.899**	0.005
TOWRE non-words	**0.811**	−0.117

*Faor loadings for each of the cognitive processing measures. Factor loadings >0.4 are highlighted in bold font*.

An initial PCA on the four temporal processing measures (TMTF cut-off frequency, AM efficiency, FM, and gap detection thresholds) suggested the extraction of three PCs; the two AM detection measures grouped together, but the FM and gap detection scores loaded significantly onto separate components (see Table 3). Since the latter two components were dominated by a single temporal processing measure, the raw FM and gap detection thresholds were entered into the regression model instead. A subsequent PCA was performed on the two AM detection measures (TMTF cut-off frequency and AM efficiency), which resulted in the extraction of a single component that explained 66% of the variance in the AM detection data (Table 3).

**Table 3:** **PCA factor loadings: Temporal processing**.

**PCA across all measures**	**PC1**	**PC2**	**PC3**
FM detection	0.004	−0.068	**0.968**
Gap detection	0.011	**0.953**	−0.076
TMTF cutoff frequency	**0.797**	−0.360	−0.295
AM efficiency	**0.824**	0.332	0.264
**PCA across AM measures**	**PC AM detection**		
TMTF cutoff frequency	**0.811**		
AM efficiency	**0.811**		

*Factor loadings for each of the temporal processing measures (top) and for the amplitude-modulation detection measures only (bottom). Factor loadings >0.4 are highlighted in bold font*.

#### 3.6.2. Regression

Following data reduction, the seven possible predictors that were entered into the regression models were; age group, PTA across 0.5–4 kHz, PC linguistic closure, PC memory and processing speed, PC AM detection, FM detection, and gap detection. Note that while individual differences in audiometric thresholds above 4 kHz could also have contributed to differences in SRTs, especially since the stimuli were filtered with a relatively shallow filter, a PTA across 6–8 kHz was not included in the regression models as a possible predictor. This is because the NAL-shaping that was applied for some older adults from 6 kHz upwards means the audiometric thresholds do not accurately reflect audibility differences in this region. Best susbsets linear regressions were performed for SRTs in babble and noise (averaged across AM and SS) separately. The final models were selected based on the Bayesian Information Criterion (BIC; Schwarz, 1978).

The analyses indicated that SRTs in babble were best predicted by PTA across 0.5–4 kHz and FM detection thresholds [*R*^2^ = 0.32, *F*_(2, 35)_ = 8.3, *p* = 0.001; see Table 4]. Thus, age-related cognitive declines in working memory and processing speed did not in fact predict SRTs in babble. Instead, when audiometric thresholds were accounted for, FM detection thresholds were the primary predictor of SRTs in babble. This would imply that TFS processing in part determines speech understanding in the presence of competing talkers.

**Table 4:** **Best subsets regression**.

**Dependent variable**	**Predictors**	***b***	**β**	***SE***	***p***	***R***^**2**^ **change**
SRT babble	PTA 0.5–4 kHz	0.17	0.51	0.047	<0.001^***^	0.2
	FM	1.57	0.35	0.64	0.02^*^	0.12
SRT noise maskers	PTA 0.5–4 kHz	0.09	0.31	0.045	0.045^*^	0.06
	Linguistic closure	0.59	0.4	0.21	0.008^**^	0.16
	Memory and processing speed	0.5	0.34	0.22	0.03^*^	0.1

Results of the best subsets regression analyses on SRTs in babble and the average SRTs across the two noise maskers (i.e., AM and SS noise;

* *significant at α = 0.05*,

** *significant at α = 0.01*,

*** *significant at α = 0.001)*.

*Note that β refers to the standardized regression coefficient. The R^2^ change reflects the proportion of the variance accounted for as predictors are added to the model*.

SRTs in noise, by contrast, were best predicted by a model with PTA across 0.5–4 kHz, linguistic closure, and memory and processing speed [*R*^2^ = 0.32, *F*_(3, 34)_ = 5.48, *p* = 0.004; see Table 4]. The fact that, after controlling for audiometric thresholds, the two cognitive measures, rather than FM detection thresholds, were significant predictors of SRTs in noise suggests that TFS processing might be less important for the perception of speech in noise maskers than in the periodic two-talker babble.

While the results from the best subsets regression analyses appear to suggest that the underlying processes accounting for individual differences in speech perception in two-talker babble and noise maskers is different, this may in fact not be the case. Even though the regression coefficients may be significant in one model but not the other, these differences in significance are in themselves not necessarily significant (Gelman and Stern, 2006). To assess whether the slopes of the predictors in the two models were indeed significantly different, a linear regression with the four predictors that were significant in either of the two best subsets regression models (PTA 0.5–4 kHZ, FM, PC linguistic closure, PC memory and processing speed) was performed on both SRTs in babble and noise separately (see Table 5). The results of this regression model are in line with the results of the best subsets regressions, with the same predictors coming out as significant [SRT babble: *R*^2^ = 0.36, *F*_(4, 33)_ = 4.7, *p* = 0.004; SRT noise maskers: *R*^2^ = 0.37, *F*_(4, 33)_ = 4.839, *p* = 0.003]. Since both models now contained the same predictors, the regression coefficients could be compared. In order to do so, a subsequent linear regression was conducted on both SRTs, with an additional dummy-coded predictor indicating the type of background noise (i.e., babble or noise maskers). The interaction between the dummy variable and the original predictors indicated whether the slopes of the predictors differed depending on the type of background noise. The results did not reveal any significant interactions (see Table 5), suggesting that even though some measures significantly predicted SRTs in one type of background noise but not the other, the regression coefficients across the models were themselves not significantly different. In other words, there is no support for the claim that the underlying processes involved in the perception of speech in babble and noise maskers are different.

**Table 5:** **Full regression model**.

**Dependent variable**	**Predictors**	***b***	**β**	***SE***	***p***	***R***^**2**^ **change**
SRT babble	PTA 0.5–4 kHz	0.16	0.49	0.05	0.003^**^	0.2
	Linguistic closure	0.35	0.22	0.23	0.14	0.06
	Memory and processing speed	0.001	0.0008	0.26	0.99	0.02
	FM	1.46	0.32	0.7	0.045^*^	0.08
SRT noise maskers	PTA 0.5–4 kHz	0.115	0.379	0.046	0.02^*^	0.06
	Linguistic closure	0.55	0.37	0.21	0.01^*^	0.16
	Memory and processing speed	0.65	0.44	0.24	0.01^*^	0.1
	FM	0.97	0.24	0.64	0.14	0.04
Interaction with SRT	PTA 0.5–4 kHz	0.023	0.1	0.034	0.5	0.005
	Linguistic closure	−0.1	−0.038	0.16	0.53	0.001
	Memory and processing speed	−0.33	−0.12	0.18	0.07	0.017
	FM	0.24	0.1	0.48	0.61	0.001

Top: Results of the regression analyses on the SRTs in babble and the average SRTs across the two noise maskers (i.e., AM and SS noise) with the four predictors that were significant in either of the two best subsets regression models (PTA 0.5–4 kHZ, FM, PC linguistic closure, PC memory and processing speed). Bottom: Results of the regression analysis on both SRT measures with an additional dummy-coded predictor indicating the type of background noise (i.e., babble or noise maskers) assessing whether the slopes of the predictors differed depending on the type of background noise. Significant results are highlighted in bold font (

* *significant at α = 0.05*,

** *significant at α = 0.01)*.

*Note that β refers to the standardized regression coefficient. The R^2^ change reflects the proportion of the variance accounted for as predictors are added to the model*.

## 4. Discussion

The aim of this study was to assess why older adults, even in the absence of hearing impairment, typically experience increased difficulties understanding speech in noise. These difficulties are typically attributed to an age-related decline in central auditory processing, particularly in the time domain, and/or a decline in cognitive function (CHABA, 1988). This study examined the relative contribution of age-related declines in both auditory temporal and cognitive processing on the perception of speech in the presence of different noise maskers.

First, it is important to note that the data in fact suggest that older adults with fairly good hearing do not necessarily perform more poorly on a speech in noise task when ecologically valid stimuli are used. Group differences were found only in the presence of two-talker babble but not in steady-state (SS) or fluctuating (AM) noise maskers. These findings are in line with the idea that competing speech is particularly detrimental for older adults (Tun and Wingfield, 1999; Helfer and Freyman, 2008; Rajan and Cainer, 2008). The fact that the older adults performed more poorly only in the presence of two-talker babble, but not the two noise maskers, suggests that these difficulties may be due to increased susceptibility to informational masking (c.f. Freyman et al., 2004). However, it may similarly be attributable to a reduced ability to make use of periodicity cues to successfully segregate the target and masker.

Contrary to expectations, the data suggest that normal hearing older adults do not have reduced glimpsing abilities (c.f. Stuart and Phillips, 1996; Peters et al., 1998; Dubno et al., 2002, 2003; Gifford et al., 2007; Grose et al., 2009). It should be noted, however, that the idea that older adults have impaired glimpsing abilities is perhaps somewhat controversial since age-related declines in FMB reported in the literature may in part have been the result of group differences that also became apparent in SS noise (c.f. Stuart and Phillips, 1996; Dubno et al., 2002, 2003; Bernstein and Grant, 2009).

It is perhaps surprising that the older adults did not perform more poorly on the speech in noise task compared to the younger listeners. One might argue that the tasks were not challenging enough. However, it is important to remember that the task was adaptive and therefore always got difficult. Moreover, while studies in the past have often used simple stimuli, such as syllables (e.g., Stuart and Phillips, 1996; Dubno et al., 2002, 2003) or simple BKB or HINT sentences (e.g., Gifford et al., 2007; Rajan and Cainer, 2008), this study used the more challenging IEEE sentences (see also Grose et al., 2009). It should be noted, however, that it remains possible that the older adults had to expend greater listening effort to perform on a par with the younger listeners.

Given that older adults are relatively unimpaired in their perception of speech in noise, could it be that the older adults are similarly unimpaired in terms of auditory temporal and cognitive processing? While an age-related decline in temporal auditory processing is well documented in normal-hearing older adults (e.g., CHABA, 1988; Frisina and Frisina, 1997; Pichora-Fuller and Souza, 2003; Gordon-Salant, 2005; Pichora-Fuller et al., 2007), this study found no decline in either ENV or TFS processing. However, the fact that AM detection thresholds were not different between young and older adults is likely because age effects only become apparent at higher modulation rates than those assessed in the present study (above about 200 Hz, Purcell et al., 2004; Grose et al., 2009). Furthermore, the lack of an age-related increase in gap detection thresholds may be related to the temporal location of the gap. He et al. (1999) only found large age-related declines when the gap was located close to the stimulus onset or offset (at 5 or 95% of the stimulus duration), and when the gap location was random from trial to trial. Consistent with our findings, gaps in the central region of a noise burst were equally detectable by younger and older listeners, even when randomly located. Whatever the exact nature of the deficit in the older listeners found by He et al. (1999) is, it is certainly not a simple deficit in ENV processing. Instead, the importance of gap uncertainty suggests a cognitive component. What is perhaps most surprising is the absence of a decline in TFS processing as this has been found using a variety of psychophysical measures (He et al., 2007; Grose and Mamo, 2010; Füllgrabe, 2013). While aging has been shown to negatively affect frequency modulation (FM) detection using low carrier frequencies (≤4 kHz) and low modulation rates (≤5 Hz) (He et al., 2007), which is thought to be primarily dependent on the neural phase-locking (Moore and Sek, 1995, 1996), we did not replicate this finding.

Similarly, aging has often been associated with declines in cognitive abilities thought to be important for the perception of speech in noise, such as working memory, attention, and processing speed (Craik and Byrd, 1982; Kausler, 1994; Salthouse, 1996). The current data indeed show declines in both working memory and processing speed. By contrast, however, attentional switching, as measured by the Visual Elevator task (Robertson et al., 1996), was not affected by age. This is somewhat surprising since this task is thought to be similar to the Wisconsin Card Sorting Test (Nelson, 1976; Robertson et al., 1996), which has repeatedly been shown to be negatively affected by age (Rhodes, 2004). Another factor thought to be important for the perception of speech in noise is linguistic closure, which was assessed by the TRT task (Zekveld et al., 2007). The literature is inconclusive as to whether linguistic closure is negatively affected by age. The results from the present study suggest that older adults do not have problems reconstructing partially masked text. This may be because linguistic closure is representative of crystallized intelligence, which does not decline with age, as opposed to fluid intelligence, which does decline with age (Horn and Cattell, 1967).

It should be noted that the absence of any age-related declines in attention, linguistic closure, and perhaps even auditory temporal processing, could in part be attributed to the fact that the older adults who participated in this study were exceptional, if only in the sense that they had good hearing. Given that cognitive declines have been linked to hearing loss (c.f. Lin et al., 2013), it may not be surprising that the normal hearing older adults who participated in this study were relatively unimpaired in the cognitive domain. This means, however, that while this study may tell us something about normal hearing older adults, the findings cannot be generalized to a more typical hearing impaired older population.

Despite the declines in working memory and processing speed, normal hearing older adults did not have increased difficulties understanding speech in SS and AM noise. This suggests that cognitive declines associated with aging do not inevitably lead to speech in noise problems. Furthermore, while the older adults performed worse on the speech perception task in the presence of two-talker babble, this could not be explained by age-related cognitive declines in working memory or processing speed when accounting for differences in audiometric thresholds. This lack of association may in part be attributed by the fact that the inter-individual variability in the data set was relatively small. Instead, however, individual differences in SRTs in babble were best predicted by audiometric thresholds and TFS processing, as measured by the FM detection task. It should be noted, however, that since the older adults had higher audiometric thresholds, it is difficult to distinguish between an explanation based on age, and one based on hearing status. The fact that TFS processing, second to audiometric thresholds, was predictive of speech perception in the presence of competing talkers suggests that variability in performance was largely due to differences in abilities to use periodicity cues. However, whether the difficulties in the presence of babble are in fact due to a reduced ability to use periodicity cues in the masker, informational masking, or even reduced glimpsing abilities remains unclear.

While it is tempting to conclude that the underlying processes involved in the perception of speech in babble and noise maskers is different, the current study did not provide sufficient support for this idea. In fact, TFS processing may be equally important for the perception of speech in noise maskers as in the presence of competing speech. Similarly, while cognitive processing was found to be predictive of SRTs in noise maskers, they may be equally important in the presence of babble. Since the predictor coefficients across the two regression models (SRTs in babble and noise maskers) were not significantly different, no conclusions can be drawn regarding differences in underlying processes involved in speech perception in the two interferer types.

In sum, this study set out to determine the relative contribution of age-related declines in auditory temporal and cognitive processing on the perception of speech in different maskers for normal-hearing older adults. The findings can be summarized as follows:

1. Older adults meeting a relatively stringent criterion for normal hearing experienced increased difficulties understanding speech only in the presence of two-talker babble.
2. Glimpsing abilities in 10-Hz sinusoidal amplitude-modulated noise were not reduced for the older adults.
3. While age-related declines in temporal auditory processing are well documented for older adults, even in the absence of hearing loss, this study failed to identify a decline in either envelope or temporal fine structure processing.
4. Older adults showed cognitive declines in working memory capacity and processing speed. Despite these declines, however, speech perception in steady-state and amplitude-modulated noise was not impaired. Moreover, reduced working memory capacity and processing speed could not explain SRTs in babble beyond differences in audiometric thresholds.

## Author contributions

This work is part of Tim Schoof's PhD project, supervised by Stuart Rosen.

## Funding

This work was supported by a PhD studentship grant funded jointly by Action on Hearing Loss and Age UK (grant S19).

### Conflict of interest statement

The authors declare that the research was conducted in the absence of any commercial or financial relationships that could be construed as a potential conflict of interest.
